# Network Pharmacology Guided Drug Repurposing and Molecular Modeling Identify Sulfasalazine as a Potential OXA-23 β-Lactamase in Carbapenem-Resistant *Acinetobacter baumannii*

**DOI:** 10.3390/ijms27146390

**Published:** 2026-07-18

**Authors:** Hanan Abdulrahman Sagini

**Affiliations:** Department of Biological Sciences, College of Sciences, University of Jeddah, Jeddah 21959, Saudi Arabia; hsajini@uj.edu.sa

**Keywords:** drug repurposing, network pharmacology, molecular dynamics simulation, OXA-23 β-lactamase, *Acinetobacter baumannii*

## Abstract

The rapid emergence of carbapenem-resistant *Acinetobacter baumannii* has become a major health concern, primarily driven by the dissemination of class D β-lactamases, particularly OXA-23, which compromise the efficacy of last-line β-lactam antibiotics. Drug repurposing combined with structure-based computational approaches provides a promising strategy for accelerating the discovery of novel therapeutic candidates against multidrug-resistant pathogens. This study aimed to identify FDA-approved non-steroidal anti-inflammatory drugs (NSAIDS) with potential inhibitory activity against OXA-23 β-lactamase by using a comprehensive computational drug discovery workflow. Twenty-six FDA-approved NSAIDs were evaluated using an integrated computational pipeline comprising network pharmacology, KEGG pathway analysis, molecular docking, molecular dynamics simulations and ADMET profiling. KEGG pathway analysis confirmed the central role of OXA-23 in β-lactam resistance, while network pharmacology prioritized nine candidates NSAIDS for subsequent structure-based investigation. Molecular docking was performed using the crystal structure of OXA-23 β-lactamase (PDB ID: 4K0X), followed by molecular dynamics simulations to assess the stability of the protein–ligand complexes. Among the prioritized compounds, sulfasalazine demonstrated the most favorable predicted binding affinity (−8.3 kcal/mol), forming stable interactions with key catalytic residues, including SER126, VAL128, and LEU166 and exhibiting a more favorable docking profile than the reference drug imipenem (−5.7 kcal/mol). Molecular dynamics simulations supported the structural stability of the sulfasalazine OXA-23 complex throughout the simulation period. Furthermore, ADMET analysis indicated favorable pharmacokinetic characteristics including good oral bioavailability, high gastrointestinal absorption, low central nervous system penetration, and an acceptable predicted safety profile. This integrated computational study identifies sulfasalazine as a promising repurposing candidate for targeting OXA-23 β-lactamase in carbapenem-resistant *A. baumannii*. The findings demonstrate the utility of combining network pharmacology with molecular modeling to prioritize candidate therapeutics and provide a computational framework for accelerating antimicrobial drug discovery. Experimental validation is warranted to confirm the inhibitory activity and therapeutic potential of sulfasalazine against multidrug-resistant *A. baumannii*.

## 1. Introduction

Antimicrobial resistance (AMR) has emerged as one of the most pressing global public health challenges of the 21st century, threatening the effectiveness of existing antibiotics and substantially increasing morbidity, mortality and healthcare costs. The rapid dissemination of multidrug-resistant bacterial pathogens has severely limited available treatment options, highlighting the urgent need for innovative and cost-effective strategies to identify new antimicrobial agents [1,2].

Carbapenem-resistant *Acinetobacter baumannii* has been a global concern that raises a significant threat to public health, largely driven by the expression of class D β-lactamases, particularly *blaOXA-23*. Current treatment options are increasingly ineffective, necessitating alternative therapeutic strategies. *Acinetobacter baumannii* has emerged as one of the most notorious multidrug-resistant (MDR) pathogens in healthcare settings, particularly in intensive care units (ICUs) [1]. This Gram-negative opportunistic bacterium is responsible for a broad range of nosocomial infections, including ventilator-associated pneumonia, bloodstream infections, urinary tract infections, wound infections, and meningitis [2]. Its pathogenicity is reinforced by multiple virulence factors, such as outer membrane proteins, biofilm formation, and iron acquisition systems, which enhance its survival in hostile environments and promote resistance against both immune responses and antibiotic treatment [3]. The organism’s ability to persist on hospital surfaces and medical equipment for prolonged periods contributes significantly to its high transmission rate. Due to its rapid acquisition of resistance mechanisms and robust survival strategies, *A. baumannii* is recognized as a critical threat to public health by the World Health Organization (WHO) [4]. The treatment of *A. baumannii* infections has historically relied on broad-spectrum antibiotics, particularly carbapenems, cephalosporins, aminoglycosides, and polymyxins. Among these, carbapenems like imipenem and meropenem were considered last-resort drugs for treating severe *A. baumannii* infections [5]. However, over the past decade, the emergence of carbapenem-resistant *A. baumannii* (CRAB) strains has drastically limited therapeutic options. Resistance has primarily developed through enzymatic degradation (β-lactamases), efflux pump overexpression, and porin mutations [6]. The rise in extensively drug-resistant (XDR) and pan-drug-resistant (PDR) strains has rendered many conventional antibiotics ineffective, necessitating the urgent development of novel antimicrobial agents or innovative therapeutic strategies that can bypass current resistance mechanisms [7].

One of the most critical factors driving carbapenem resistance in *A. baumannii* is the expression of the *blaOXA-23* gene, which encodes a class D β-lactamase enzyme. The OXA-23 enzyme hydrolyzes β-lactam antibiotics, including carbapenems, by breaking their β-lactam ring, thus nullifying their bactericidal effect [8]. Unlike many β-lactamases, OXA-23 has a broad substrate profile and is often associated with mobile genetic elements such as plasmids and transposons, facilitating its horizontal gene transfer among bacterial populations [9]. The widespread distribution and genetic plasticity of *blaOXA-23* make it a significant biomarker for resistance surveillance and a priority target for therapeutic intervention [10]. Inhibiting the function of this enzyme is therefore considered a strategic approach to restoring the efficacy of carbapenem antibiotics and controlling MDR *A. baumannii* outbreaks [11]. Drug repurposing, the strategy of identifying new therapeutic uses for existing drugs, offers a time-efficient and cost-effective alternative to conventional drug discovery. Similar to previous repurposing approaches, such as the computational framework reported by Kulkarni et al., which integrated drug–target interactions and molecular modeling to identify novel therapeutic candidates, the present study applies a systematic in silico repurposing strategy [12]. It is particularly valuable in addressing antimicrobial resistance, where the development pipeline for new antibiotics remains limited. Among repurposed candidates, non-steroidal anti-inflammatory drugs (NSAIDs) have shown potential antimicrobial and anti-biofilm activity in addition to their known anti-inflammatory effects [13]. Unlike conventional antibiotics, NSAIDs are not structurally similar to β-lactams, which reduces the likelihood of existing resistance mechanisms targeting them. Repurposing FDA-approved NSAIDs against *blaOXA-23* presents a promising strategy to overcome carbapenem resistance, as these drugs already possess well-established safety, pharmacokinetic, and toxicity profiles, potentially accelerating their clinical deployment [14]. This dual activity—anti-inflammatory and antibacterial—could offer synergistic benefits in managing severe *A. baumannii* infections, especially in immunocompromised or critically ill patients [15].

This study aims to identify FDA-approved NSAIDs with the potential to inhibit the *blaOXA-23* enzyme in *Acinetobacter baumannii* through a network pharmacology-guided drug repurposing approach. The methodology begins with the retrieval of the 3D structure of *blaOXA-23*, followed by KEGG pathway analysis to understand its resistance mechanism. A comprehensive dataset of 26 FDA-approved NSAIDs was curated, and their potential interactions with *blaOXA-23* were explored through network pharmacological analysis using Cytoscape. Topologically significant drugs were selected for molecular docking using PyRx to evaluate binding affinity and interaction profiles. The most promising candidate was compared with imipenem to determine relative binding efficiency. Molecular dynamics (MD) simulations were conducted using GROMACS 2026.2 to assess the stability and conformational behavior of the drug–protein complex. Finally, ADMET profiling and toxicity prediction were performed using SwissADME and ProTox-II, respectively, to evaluate the pharmacokinetic suitability and safety of the best repurposed NSAID candidate. This integrated in silico framework offers a systematic and robust approach to identifying novel therapeutic options against carbapenem-resistant *A. baumannii*.

## 2. Results

### 2.1. Retrieval of blaOXA-23 Protein

**Figure 1:**
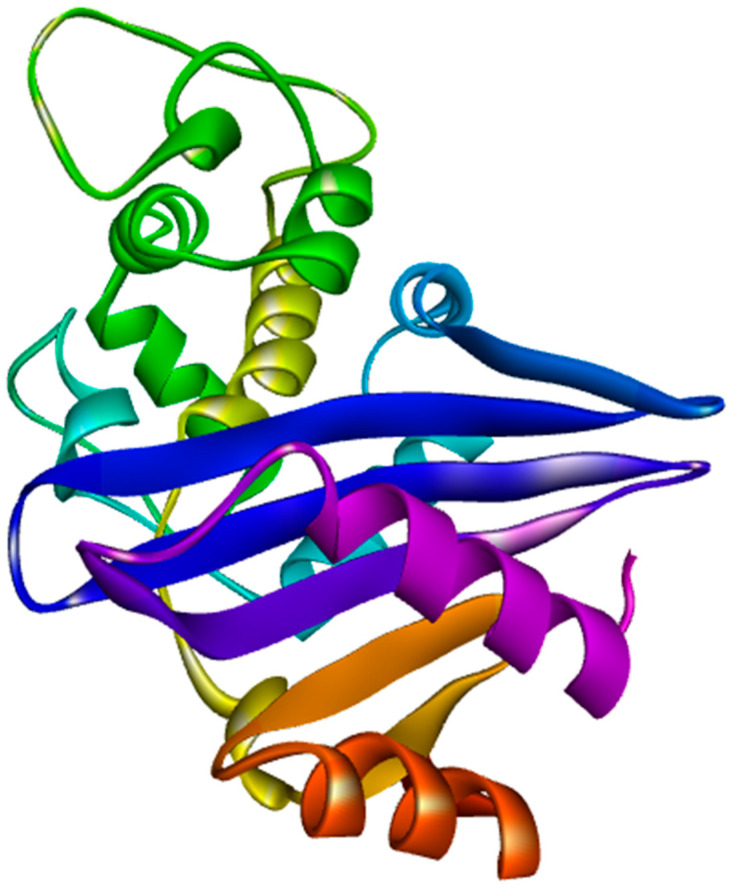
Three-dimensional ribbon representation of the *blaOXA-23* protein structure retrieved from the Protein Data Bank (PDB ID: 4K0X), showing the spatial arrangement of α-helices and β-sheets in a rainbow color gradient from N-terminus (blue) to C-terminus (red).

The three-dimensional (3D) crystal structure of the *blaOXA-23* β-lactamase enzyme from *Acinetobacter baumannii* was successfully retrieved from the Protein Data Bank (PDB) under the accession ID 4K0X (Figure 1). The structure was resolved by X-ray crystallography at a high resolution of 1.61 Å, offering detailed atomic insights necessary for structure-based drug discovery. The protein comprises multiple α-helices and β-sheets arranged in a compact globular form, characteristic of class D β-lactamases. This structural information served as a foundation for downstream computational analyses to identify potential inhibitors targeting carbapenem resistance.

### 2.2. Pathway Analysis of blaOXA-23

**Figure 2:**
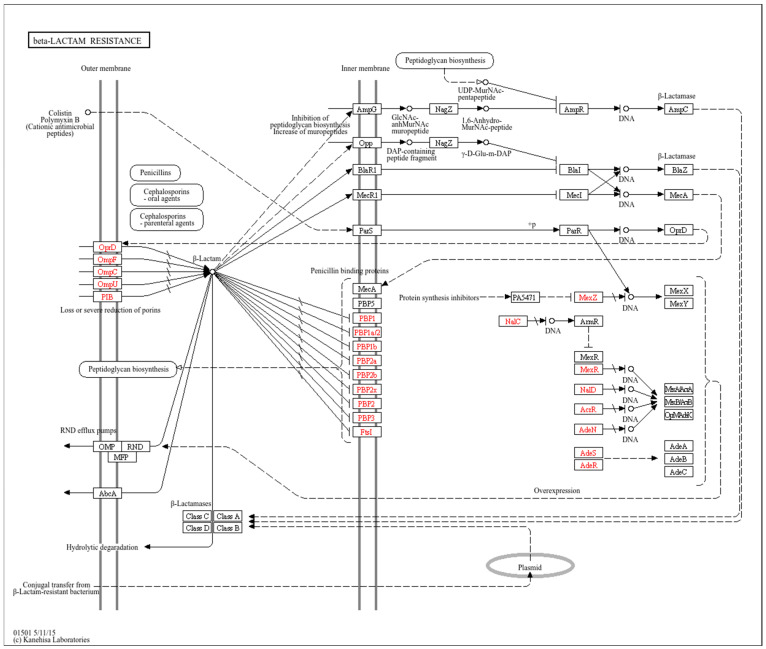
KEGG β-lactam resistance pathway (map01501) highlighting the involvement of *blaOXA-23* (Class D β-lactamase, K18793) in the enzymatic inactivation of carbapenems. The pathway also illustrates other resistance mechanisms such as porin modification, efflux pump expression, and penicillin-binding protein alterations.

The β-lactam resistance pathway (Figure 2) in *Acinetobacter baumannii* represents a complex and multi-tiered defense mechanism that allows the bacterium to survive exposure to β-lactam antibiotics, including carbapenems, penicillins, and cephalosporins. The pathway begins at the outer membrane, where reduced expression or complete loss of porin proteins such as OmpF and OmpC limits the entry of antibiotics into the periplasmic space, effectively lowering the intracellular concentration of the drug. This is further complemented by the activation of Resistance-Nodulation-Division (RND) family efflux pumps that actively expel β-lactam antibiotics from the bacterial cell, thereby preventing them from reaching their targets. Within the periplasm, one of the key targets of β-lactam antibiotics is the penicillin-binding proteins (PBPs), which are essential for bacterial cell wall synthesis. However, structural modifications in PBPs, such as PBP1a, PBP2, and PBP3, reduce the binding affinity of antibiotics, diminishing their inhibitory effect. The most crucial component of this resistance network is the production of β-lactamase enzymes, particularly Class D β-lactamases like *blaOXA-23*. These enzymes hydrolyze the β-lactam ring, a critical structural feature of β-lactam antibiotics, rendering the drugs inactive before they can exert their antibacterial effect.

The pathway is regulated at the genetic level by various regulatory proteins and mobile genetic elements, which can enhance the expression of resistance genes or facilitate their horizontal transfer between bacterial strains. Collectively, this integrated resistance system allows *A. baumannii* to withstand even the most potent β-lactam antibiotics, including carbapenems, and establishes *blaOXA-23* as a central player in mediating carbapenem resistance. This highlights the enzyme’s significance as a therapeutic target in combating multidrug-resistant infections.

### 2.3. Retrieval of FDA-Approved NSAIDs

A total of 26 FDA-approved non-steroidal anti-inflammatory drugs (NSAIDs) were retrieved through a systematic search in the DrugBank database. This list was compiled from an initial pool of 132 NSAIDs, narrowed down based on FDA approval status. The corresponding 3D structures of the shortlisted drugs were obtained from the PubChem database in SDF format, enabling their use in downstream computational screening against the *blaOXA-23* enzyme of *Acinetobacter baumannii*.

These drugs included a wide range of chemical classes and structural frameworks, offering diverse molecular scaffolds for docking and interaction studies. The retrieved FDA-approved NSAIDs are Aspirin, Balsalazide, Bromfenac, Celecoxib, Diclofenac, Diflunisal, Etodolac, Fenoprofen, Flurbiprofen, Ibuprofen, Indomethacin, Ketoprofen, Ketorolac, Meclofenamic acid, Mefenamic acid, Meloxicam, Nabumetone, Naproxen, Nepafenac, Oxaprozin, Piroxicam, Sulfasalazine, Sulindac, Suprofen, Tolmetin, and Valdecoxib. Each drug was characterized in terms of its DrugBank ID, PubChem CID, molecular formula, molecular weight, and SMILES notation (Table 1). Their physicochemical diversity was considered advantageous in identifying potential repurposing candidates with favorable interaction profiles against the *blaOXA-23* protein. These 26 NSAIDs served as the foundation for further pharmacological network analysis, molecular docking, and in silico ADMET and toxicity assessments to explore their repositioning as antibacterial agents targeting carbapenem-resistant *A. baumannii*.

### 2.4. Network Pharmacological Analysis

**Figure 3:**
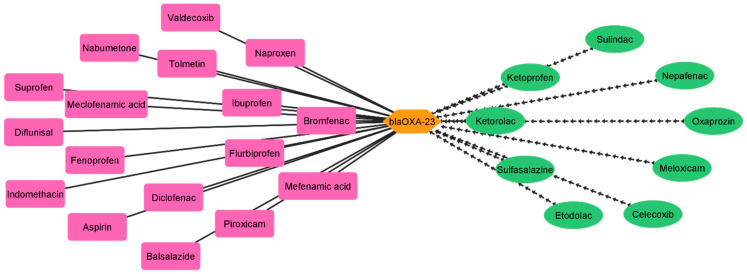
Network pharmacology map showing interactions between FDA-approved NSAIDs and *blaOXA-23* enzyme. Green nodes represent drugs with strong network interaction parameters; pink nodes indicate moderate to weak interactors; the central orange node represents the *blaOXA-23* target.

Network pharmacological analysis was conducted using Cytoscape (v3.9.1) to evaluate the interaction between *blaOXA-23* and 26 FDA-approved NSAIDs. The resulting drug–target interaction network consisted of 27 nodes and 26 edges, with an average number of neighbors of 1.9. The analysis was completed in 0.58 s. Topological parameters such as degree centrality, betweenness centrality, closeness centrality, and edge score were used to assess the importance of each drug within the network (Figure 3). Drugs that exhibited strong network connections were those with degree centrality ≥10, betweenness centrality ≥0.05, closeness centrality ≥0.5, and edge scores ≥0.80. Based on these criteria, nine NSAIDs showed close interaction with *blaOXA-23*. These drugs—Ketoprofen, Sulindac, Ketorolac, Nepafenac, Oxaprozin, Meloxicam, Celecoxib, Sulfasalazine, and Etodolac—were identified as the most promising candidates and were highlighted in green within the network.

In contrast, the remaining 17 drugs, including widely used agents like Ibuprofen, Aspirin, Naproxen, and others, exhibited lower topological parameter values, falling into the moderate or weak interaction category and were visually marked in pink. The *blaOXA-23* protein, serving as the central target, was highlighted in orange. These results suggest that the nine shortlisted NSAIDs have strong interaction potential with *blaOXA-23*, making them suitable for further validation. Consequently, these nine drugs were selected for subsequent molecular docking analysis to explore their binding affinity and potential as repurposed inhibitors against carbapenem-resistant *Acinetobacter baumannii*.

### 2.5. Molecular Docking Analysis

**Figure 4:**
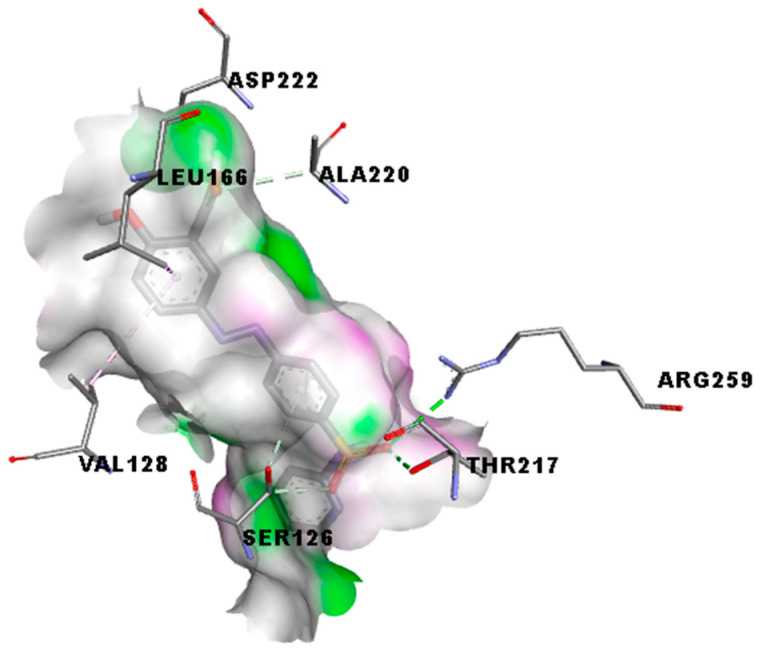
Molecular docking interaction of sulfasalazine with the active site of *blaOXA-23*, showing involvement of key residues (SER126, VAL128, LEU166, THR217, ALA220, ASP222, ARG259) and various binding interactions including hydrogen bonding and π-type interactions.

Molecular docking analysis was performed on the nine FDA-approved NSAIDs shortlisted through network pharmacology to evaluate their binding affinity and interaction profile with the *blaOXA-23* enzyme (PDB ID: 4K0X). Using AutoDock Vina 1.1.2 through PyRx, each drug was docked within the active site of the protein, and the binding affinities were calculated in kcal/mol. Among the tested drugs, Sulfasalazine showed the highest binding affinity with a docking score of −8.3 kcal/mol, indicating a strong and stable interaction with *blaOXA-23*. It formed critical molecular interactions with key active site residues including SER126, VAL128, LEU166, THR217, ALA220, ASP222, and ARG259. The types of interactions observed involved conventional hydrogen bonds, π-donor hydrogen bonds, π-sigma, π-alkyl, and carbon-hydrogen bonds, suggesting a multifaceted binding mechanism that may enhance its inhibitory potential (Figure 4).

Other drugs that demonstrated strong binding affinities included Sulindac (−8.1 kcal/mol), Nepafenac (−8.0 kcal/mol), Meloxicam (−7.8 kcal/mol), and Oxaprozin (−7.7 kcal/mol). Ketoprofen and Celecoxib both exhibited docking scores of −7.5 kcal/mol, while Etodolac and Ketorolac showed slightly lower, yet still significant, affinities of −7.4 and −7.1 kcal/mol, respectively. All nine drugs fell within the range of strong to moderate binding thresholds (≤−7.0 kcal/mol), validating their potential as repurposed inhibitors targeting *blaOXA-23* in carbapenem-resistant *Acinetobacter baumannii*. These results not only highlight the therapeutic promise of Sulfasalazine but also establish a comparative framework for evaluating NSAIDs as viable candidates in overcoming multidrug resistance through non-antibiotic mechanisms.

### 2.6. Comparative Analysis with Carbapenem

**Figure 5:**
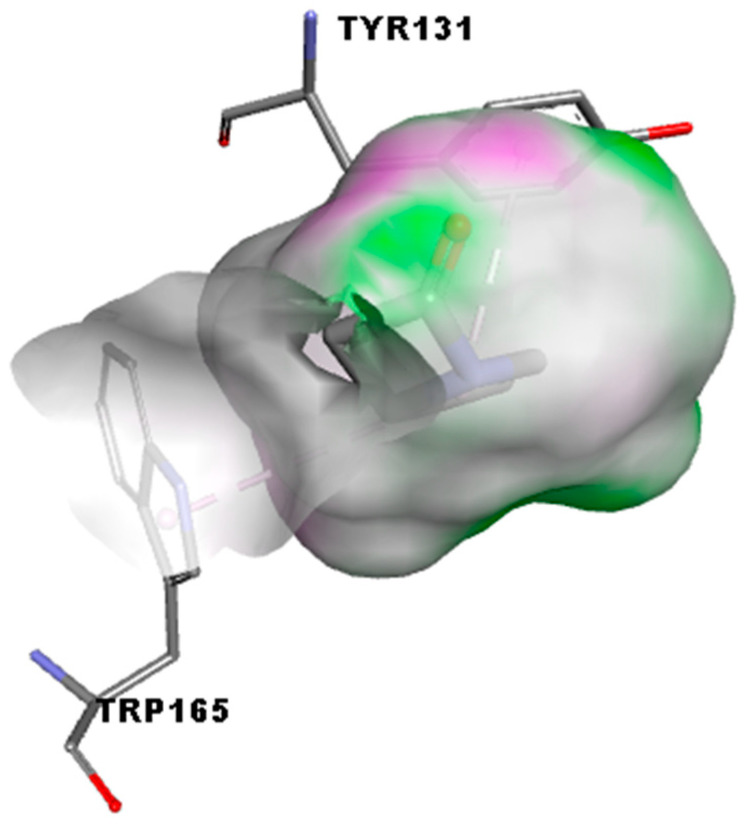
Molecular docking interaction of imipenem with *blaOXA-23* enzyme, showing weak binding affinity (−5.7 kcal/mol) and limited interaction with residues TYR131 and TRP165 through π-alkyl bonding.

A comparative molecular docking analysis was to validate the inhibitory potential of the top-performing NSAID, sulfasalazine, a comparative molecular docking analysis was conducted with imipenem—a representative carbapenem antibiotic widely used against *Acinetobacter baumannii*. The docking results revealed a binding affinity of −5.7 kcal/mol (Figure 5) for imipenem when docked with *blaOXA-23*, significantly weaker than sulfasalazine, which demonstrated a binding affinity of −8.3 kcal/mol under identical docking conditions.

Imipenem formed limited molecular interactions with *blaOXA-23*, involving only two key residues: TYR131 and TRP165, and the primary interaction type observed was π-alkyl bonding. In contrast, sulfasalazine formed multiple strong interactions, including hydrogen bonds and π-type interactions with critical active site residues such as SER126, VAL128, LEU166, THR217, ALA220, ASP222, and ARG259. These results indicate a more stable and specific binding profile for sulfasalazine, underscoring its greater potential as a repurposed inhibitor against carbapenem-resistant *A. baumannii*. This comparative analysis provides structural binding strength and interaction complexity highlights of sulfasalazine’s superior potential as a repurposed inhibitor of *blaOXA-23*, compared to the conventional and now less effective carbapenem antibiotic imipenem. These findings support the repositioning of sulfasalazine as a promising candidate in the fight against carbapenem-resistant *A. baumannii* infections.

### 2.7. Molecular Dynamics Simulations Analysis

**Figure 6:**
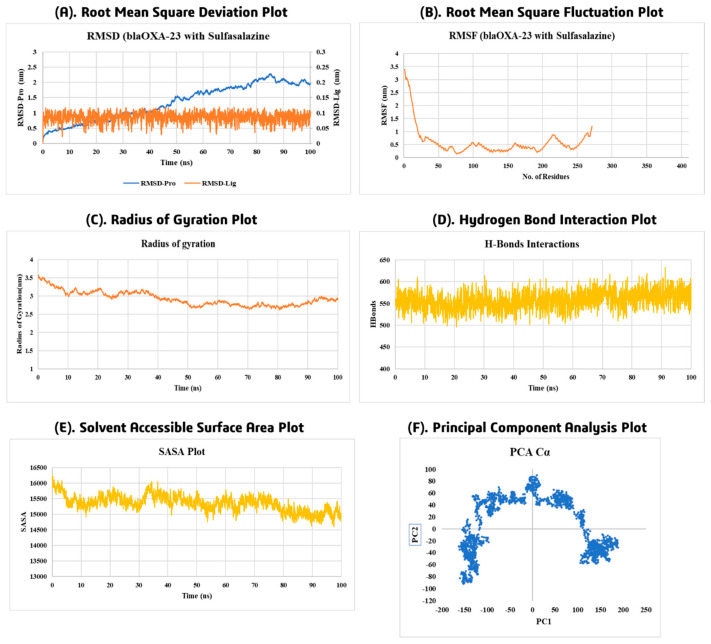
Molecular dynamics simulation plots of sulfasalazine in complex with *blaOXA-23*. (**A**) RMSD of protein and ligand over 100 ns shows complex stability. (**B**) RMSF plot showing residue-wise flexibility. (**C**) Radius of gyration indicating compactness. (**D**) Hydrogen bond analysis suggesting strong interactions. (**E**) SASA plot showing reduction in solvent exposure. (**F**) PCA plot indicating significant conformational dynamics.

The molecular dynamics (MD) simulation of sulfasalazine, a repurposed non-steroidal anti-inflammatory drug (NSAID), in complex with the *blaOXA-23* protein, was conducted to evaluate its stability and interaction profile as a potential inhibitor to combat carbapenem resistance. The Root Mean Square Deviation (RMSD) plot (Figure 6A) indicates that the protein–ligand complex achieves equilibrium over the 100 ns simulation period. The RMSD of the protein (RMSD-Pro) gradually increases and stabilizes around 2.5 nm, suggesting a stable binding conformation. The ligand (RMSD-Lig) remains consistently stable around \~0.1 nm, confirming firm binding within the active site.

The Root Mean Square Fluctuation (RMSF) analysis (Figure 6B) shows that most residues exhibit minimal fluctuations below 1.5 nm, with only a few terminal residues showing elevated flexibility beyond 2.5 nm, indicating overall structural rigidity with localized flexibility in loop regions. The Radius of Gyration (Rg) plot (Figure 6C) illustrates the compactness of the protein structure during the simulation. Rg values fluctuate between 2.8 and 3.4 nm, gradually decreasing over time, implying increased structural compactness upon ligand binding. The hydrogen bond interaction plot (Figure 6D) reveals that sulfasalazine forms consistent hydrogen bonds throughout the simulation, ranging from approximately 400 to 600 interactions, with some fluctuations, suggesting strong and persistent interactions with the protein target. The Solvent Accessible Surface Area (SASA) analysis (Figure 6E) shows a decreasing trend from around 16,000 Å^2^ to ~14,500 Å^2^ over the 100 ns, indicating reduced surface exposure and further evidence of structural compactness induced by ligand binding. The Principal Component Analysis (PCA) plot (Figure 6F) demonstrates significant conformational transitions along PC1 and PC2 axes, indicating the protein undergoes essential motions and adopts distinct conformational states, which are typical of functionally relevant dynamics. These collective insights support the potential of sulfasalazine to interact stably and effectively with the *blaOXA-23* protein, positioning it as a promising candidate for repurposing against carbapenem-resistant bacterial strains.

### 2.8. ADMET Analysis

**Figure 7:**
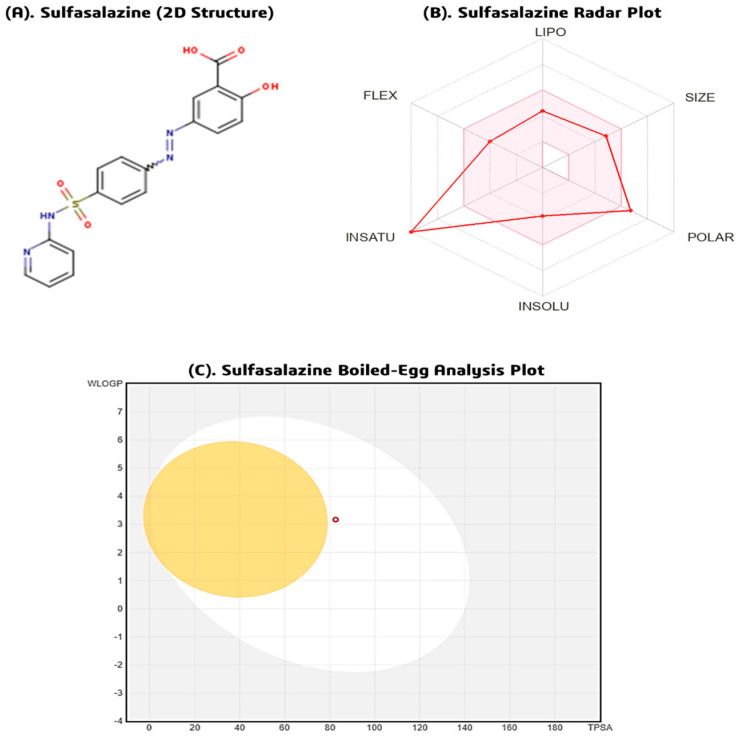
ADMET Analysis of sulfasalazine (**A**). 2D structure of sulfasalazine (**B**). Radar plot of sulfasalazine displaying physicochemical properties including lipophilicity, size, polarity, solubility, flexibility, and saturation within optimal drug-likeness ranges, indicating its suitability for oral administration (**C**). BOILED-Egg plot of sulfasalazine illustrating its predicted high gastrointestinal absorption (white region) and non-permeability across the blood–brain barrier (outside yellow yolk), supporting its use in peripheral infections such as carbapenem-resistant *A. baumannii*.

The ADMET (Absorption, Distribution, Metabolism, Excretion, and Toxicity) profiling of sulfasalazine, the top-ranked FDA-approved NSAID identified through molecular docking and network pharmacological analysis, was conducted using the SwissADME web tool (Figure 7A). This comprehensive evaluation helped in determining the drug-likeness and pharmacokinetic suitability of sulfasalazine as a repurposed therapeutic agent against *blaOXA-23*-mediated resistance in *Acinetobacter baumannii*. One of the key highlights of this analysis was the BOILED-Egg model, a predictive tool used to assess gastrointestinal (GI) absorption and blood–brain barrier (BBB) permeability (Figure 7C). In this model, sulfasalazine’s representation fell within the white region, indicating a high probability of passive gastrointestinal absorption, which is crucial for oral drug delivery. However, it was positioned outside the yellow yolk, signifying that sulfasalazine is unlikely to cross the blood–brain barrier. This is considered advantageous for antimicrobial therapy targeting peripheral infections like those caused by *A. baumannii*, as CNS penetration is not desired and would only raise the risk of central nervous system-related side effects.

Pharmacokinetic profiling (Figure 7B) showed that sulfasalazine exhibits acceptable lipophilicity, with a consensus LogP value of 2.3, falling well within the optimal range (1–3) for effective membrane permeability. Its Topological Polar Surface Area (TPSA) was calculated at 149.69 Å^2^, a value slightly above the typical threshold for BBB penetration, which aligns with its prediction of non-CNS activity, but still supports adequate oral bioavailability. The molecule showed compliance with Lipinski’s Rule of Five, having only one permissible violation due to its high polarity, which is acceptable for orally active drugs. It had 3 hydrogen bond donors, 8 acceptors, and a molar refractivity of 100.95, which supports favorable interactions with biological targets and good pharmacokinetic behavior. Regarding solubility, sulfasalazine was predicted to be moderately to highly water-soluble, with a Log S value of −3.76 as per the ESOL model. This solubility profile favors oral administration and supports systemic availability. The drug was not identified as a P-glycoprotein (P-gp) substrate, suggesting that it is less likely to be effluxed out of intestinal epithelial cells, thereby enhancing its systemic exposure and therapeutic efficacy.

From a metabolic standpoint, sulfasalazine demonstrated no inhibitory activity against major cytochrome P450 isoforms, including CYP1A2, CYP2C19, CYP2C9, CYP2D6, and CYP3A4. This reduces the likelihood of drug–drug interactions, making it a safe candidate for combination therapy. The bioavailability score of sulfasalazine was predicted to be 0.56, indicating good oral bioavailability. Additionally, the skin permeability (Log Kp) value of −7.18 cm/s indicated poor transdermal absorption, which is suitable for drugs intended for systemic, not topical, use. The radar plot generated by SwissADME also confirmed that sulfasalazine falls within the favorable zone for multiple drug-likeness criteria, including lipophilicity, molecular size, polarity, solubility, saturation, and molecular flexibility. The ADMET analysis revealed that sulfasalazine possesses a pharmacokinetic and physicochemical profile highly compatible with oral administration and systemic action. Its strong GI absorption potential, low CNS penetration, and low likelihood of metabolic interference position it as a highly promising repurposed drug candidate for the treatment of multidrug-resistant *A. baumannii* infections mediated by the *blaOXA-23* enzyme.

## 3. Discussion

Antimicrobial resistance (AMR) has become a critical global health concern, with multidrug-resistant (MDR) *Acinetobacter baumannii* representing one of the most challenging nosocomial pathogens due to its ability to acquire multiple resistance mechanisms. Among these mechanisms, the acquisition of class D β-lactamases, particularly *blaOXA-23*, is a major contributor to carbapenem resistance through efficient hydrolysis of carbapenem antibiotics [16]. Previous studies have demonstrated that *blaOXA-23* is widely disseminated among clinical *A. baumannii* isolates and is strongly associated with treatment failure, emphasizing the urgent need for alternative therapeutic strategies [17]. In contrast to conventional antibiotic discovery, which is limited by high costs and prolonged development timelines, drug repurposing provides an accelerated approach by utilizing compounds with established pharmacological and safety profiles. The present study extends this concept by exploring FDA-approved NSAIDs as potential inhibitors of *blaOXA-23* using an integrated network pharmacology and molecular modeling framework.

The use of NSAIDs as repurposing candidates was based on accumulating evidence that several members of this drug class exhibit antimicrobial activity independent of their classical cyclooxygenase inhibition. Previous reports have shown that NSAIDs such as diclofenac and ibuprofen can interfere with bacterial growth and virulence through membrane disruption, metabolic interference, and inhibition of bacterial targets [15]. Similar repurposing approaches have investigated approved drugs against bacterial resistance-associated proteins; however, most studies have focused on screening broad drug libraries or identifying antibacterial candidates through target-based approaches [18]. In the present study, we specifically evaluated NSAIDs against the clinically important resistance determinant *blaOXA-23*, providing a targeted strategy for identifying potential β-lactamase inhibitors rather than simply identifying compounds with general antibacterial activity.

The selection of the high-resolution *blaOXA-23* crystal structure (PDB ID: 4K0X; 1.61 Å resolution) enabled reliable structure-based analysis and accurate prediction of ligand interactions. The structural characteristics observed in our analysis are consistent with previous crystallographic studies describing class D β-lactamases as compact α/β-folded enzymes containing conserved catalytic residues responsible for β-lactam hydrolysis [17]. KEGG pathway analysis further supported the central role of *blaOXA-23* within the β-lactam resistance pathway, where enzymatic degradation is complemented by additional mechanisms such as altered membrane permeability and efflux activity [19]. These findings reinforce that targeting *blaOXA-23* represents a rational approach for restoring carbapenem susceptibility in resistant *A. baumannii* strains.

Network pharmacology analysis prioritized nine NSAIDs with strong interaction potential toward *blaOXA-23*, including ketoprofen, sulindac, ketorolac, nepafenac, oxaprozin, meloxicam, celecoxib, sulfasalazine, and etodolac. Similar network-based drug repurposing studies have demonstrated that integrating drug–target interaction networks with biological pathway analysis can effectively narrow large compound libraries into biologically relevant candidates [20]. However, unlike previous approaches that primarily identify drug associations based on disease networks, our study combines network prioritization with direct structural evaluation of a bacterial resistance enzyme, thereby increasing the specificity of candidate selection.

Molecular docking revealed that all prioritized NSAIDs exhibited favorable binding interactions with *blaOXA-23*, with sulfasalazine showing the strongest predicted affinity (−8.3 kcal/mol). The interaction of sulfasalazine with catalytic and neighboring residues, including SER126, THR217, VAL128, LEU166, ALA220, ASP222, and ARG259, suggests potential interference with the active-site environment required for enzymatic activity. Compared with imipenem, which demonstrated weaker binding (−5.7 kcal/mol) and fewer residue interactions, sulfasalazine showed enhanced predicted stability within the binding pocket. These observations are consistent with previous reports suggesting that non-antibiotic drugs may interact with bacterial enzymes and contribute to antimicrobial activity through mechanisms distinct from their original pharmacological roles [18,21]. Nevertheless, these computational findings require experimental confirmation to determine whether binding translates into functional inhibition of *blaOXA-23* activity.

Molecular dynamics simulation further supported the stability of the sulfasalazine–*blaOXA-23* complex, demonstrating consistent structural behavior throughout the simulation period. The stable RMSD, reduced RMSF fluctuations, and favorable Rg and SASA profiles indicate maintenance of protein–ligand integrity, consistent with previous computational studies where stable dynamic interactions were associated with stronger ligand binding potential [22]. The persistent hydrogen bonding pattern observed during simulation further suggests that sulfasalazine may maintain favorable interactions within the catalytic region of *blaOXA-23*. However, future biochemical assays are necessary to confirm enzyme inhibition and determine the precise mechanism of action.

The predicted ADMET profile of sulfasalazine further supports its potential as a repurposing candidate, showing favorable gastrointestinal absorption, limited CNS penetration, acceptable lipophilicity, and compliance with most drug-likeness parameters. Similar computational evaluations have demonstrated that repurposed drugs with established pharmacokinetic characteristics can provide valuable starting points for antimicrobial development [23]. Compared with newly discovered antibacterial molecules, sulfasalazine offers the advantage of an existing clinical history, which may reduce translational barriers during further development.

This study demonstrates the potential of combining network pharmacology, molecular docking, molecular dynamics simulation, and ADMET analysis for identifying repurposed inhibitors against antibiotic resistance mechanisms. While previous drug repurposing studies have successfully applied similar computational strategies, the present work specifically highlights NSAIDs as potential *blaOXA-23* inhibitors against carbapenem-resistant *A. baumannii*. The findings provide a computational foundation for future experimental validation, including enzymatic inhibition assays and antibacterial susceptibility studies, to evaluate the therapeutic relevance of sulfasalazine and other prioritized candidates.

## 4. Methodology

### 4.1. Retrieval of blaOXA-23 Protein of A. baumannii

The three-dimensional (3D) structure of the *blaOXA-23* β-lactamase enzyme from *Acinetobacter baumannii* was retrieved from the Protein Data Bank (PDB) (https://www.rcsb.org/) accessed on 5 April 2026 using the accession ID 4K0X. This structure was resolved through X-ray crystallography at a high resolution of 1.61 Å, ensuring detailed structural insights. The PDB file was downloaded in standard format and prepared for further computational analyses.

### 4.2. Pathway Analysis of blaOXA-23

The role of the *blaOXA-23* gene in antibiotic resistance, specifically against carbapenems, was analyzed using the Kyoto Encyclopedia of Genes and Genomes (KEGG) database. The KEGG Orthology (KO) identifier K18793, corresponding to class D β-lactamase, was searched to determine its functional association with resistance pathways. The gene was mapped to the β-lactam resistance pathway (KEGG pathway ID: map01501) and assigned to KEGG module M00851, which outlines enzymatic inactivation of β-lactam antibiotics, including carbapenems. The analysis revealed that *blaOXA-23* contributes to carbapenem resistance by hydrolyzing the antibiotic, rendering it ineffective. This pathway-based insight was obtained through the KEGG database (https://www.genome.jp/kegg/) accessed on 5 April 2026, helping to clarify the molecular mechanism of resistance and its implications in clinical treatment failure.

### 4.3. Retrieval of FDA-Approved NSAIDs

Non-steroidal anti-inflammatory drugs (NSAIDs) were retrieved and analyzed using the DrugBank database (https://go.drugbank.com/) accessed on 6 April 2026. A search for the “NSAIDs” drug class was conducted to compile a comprehensive list of compounds, resulting in the identification of 132 NSAIDs. These were further filtered based on regulatory status to extract only those approved by the U.S. Food and Drug Administration (FDA), yielding a final list of 26 FDA-approved NSAIDs. The 3D structures of these 26 FDA-approved drugs were then retrieved from the PubChem database (https://pubchem.ncbi.nlm.nih.gov/) accessed on 6 April 2026 in SDF format for further computational analysis. These structures were used in subsequent molecular docking studies to evaluate their interaction with the *blaOXA-23* target protein.

### 4.4. Network Pharmacological Analysis

Network pharmacological analysis was performed to explore the interaction between *blaOXA-23* and 26 FDA-approved NSAIDs using Cytoscape (v3.9.1) accessed on 7 April 2026. A drug–target interaction network was constructed, and several key topological parameters were assessed to determine the significance of each drug in targeting *blaOXA-23* within the context of carbapenem resistance. Degree centrality was used to measure the number of direct connections a drug had in the network; values ≥10 were considered good, 5–9 as average, and ≤4 as poor. Betweenness centrality, which reflects a drug’s control over information flow, was considered good if ≥0.05, average between 0.01 and 0.049, and poor if ≤0.009. Closeness centrality, indicating how close a drug is to all other nodes in the network, was considered effective when ≥0.5, moderate between 0.3 and 0.49, and poor if ≤0.29. The network density was also calculated, with an optimal range between 0.3 and 0.7, indicating a well-connected but not overly saturated network. Edge scores representing the strength of drug–target interactions were interpreted as strong (≥0.80), moderate (0.50–0.79), or weak (≤0.49). In addition, the MCODE plugin was employed to identify significant clusters or modules within the network, with a clustering score ≥3 and node count ≥4 considered significant. Based on these parameters, drugs with strong topological positions and high interaction scores were shortlisted as potential inhibitors of *blaOXA-23* for further validation against carbapenem-resistant *A. baumannii*.

### 4.5. Molecular Docking Analysis

Molecular docking analysis was carried out to evaluate the binding affinity of the 9 shortlisted FDA-approved NSAIDs identified through network pharmacological analysis for their potential to inhibit *blaOXA-23*. Docking studies were performed using PyRx (version 0.8), which incorporates AutoDock Vina as its docking engine accessed on 10 April 2026. The crystal structure of *blaOXA-23* (PDB ID: 4K0X) was used as the receptor, and the 3D structures of the selected drugs were prepared in SDF format and energy-minimized prior to docking. Key docking parameters included setting the grid box to enclose the active site region of *blaOXA-23*, ensuring precise ligand-receptor interactions. The exhaustiveness value was set to 8 to allow adequate sampling of binding poses. Each drug was docked multiple times to ensure reproducibility and identify the most stable binding conformation. The binding affinity (expressed in kcal/mol) was used as the primary scoring criterion, with values ≤−7.0 kcal/mol considered strong binding, −6.0 to −6.9 kcal/mol as moderate, and ≥−5.9 kcal/mol as weak. Additional analysis of docking poses included the evaluation of hydrogen bonds, hydrophobic interactions, and electrostatic contacts using Discovery Studio Visualizer 4.0. Drugs exhibiting the most favorable binding energy and interaction profile with key catalytic residues of *blaOXA-23* were prioritized as potential drug repurposing candidates to counteract carbapenem resistance.

### 4.6. Comparative Analysis with Carbapenem

To assess the inhibitory potential of the best repurposed FDA-approved NSAID against *blaOXA-23*, a comparative molecular docking analysis was performed using imipenem as a representative carbapenem antibiotic. The 3D structure of imipenem was retrieved from the PubChem database (PubChem CID: 104838) and prepared for docking. Both the top-ranked NSAID and imipenem were docked against the *blaOXA-23* protein structure (PDB ID: 4K0X) using AutoDock Vina within the PyRx platform. Docking conditions were standardized for both ligands, with the same grid box dimensions centered on the active site and an exhaustiveness value of 8 to ensure uniform sampling. The binding affinities of the compounds were calculated in kcal/mol and directly compared. Interaction profiles, including hydrogen bonding, hydrophobic interactions, and electrostatic contacts, were analyzed using Discovery Studio Visualizer to identify key residues involved in binding. This comparative analysis provided structural and energetic evidence supporting the potential repurposing of NSAIDs to combat carbapenem-resistant *A. baumannii*.

### 4.7. Molecular Dynamics Simulations Analysis

Molecular dynamics (MD) simulations were performed using GROMACS accessed on 13 April 2026 to evaluate the structural stability and dynamic behavior of the best docked *blaOXA-23*–NSAID complex. The protein–ligand complex was prepared using the GROMOS96 43a1 force field, with ligand parameters generated via PRODRG. The complex was solvated in a cubic box with the SPC/E water model, maintaining a minimum distance of 1.0 nm between the complex and the box boundaries. To neutralize the system, appropriate counter ions (Na^+^/Cl^−^) were added. Energy minimization was carried out using the steepest descent algorithm until the system reached a maximum force below 1000 kJ/mol/nm. This was followed by equilibration in two phases: a 100 ps NVT ensemble to stabilize temperature at 300 K using the Berendsen thermostat, and a 100 ps NPT ensemble to maintain pressure at 1 bar using the Parrinello–Rahman barostat. A 10 ns production run was conducted with a 2 fs time step under periodic boundary conditions.

Post-simulation analyses included calculation of Root Mean Square Deviation (RMSD) to assess complex stability, and Root Mean Square Fluctuation (RMSF) to determine flexibility of individual residues. The Radius of Gyration (Rg) was evaluated to examine the compactness of the protein structure over time, while hydrogen bonding analysis was used to monitor the interaction stability between the ligand and *blaOXA-23*. Solvent Accessible Surface Area (SASA) was calculated to understand conformational changes related to protein surface exposure. Principal Component Analysis (PCA) was also performed to explore the dominant motions and dynamic conformational transitions of the complex. Together, these parameters provided a comprehensive insight into the stability, flexibility, and binding persistence of the selected NSAID as a repurposing candidate against *blaOXA-23*.

### 4.8. ADMET Analysis

The ADMET (Absorption, Distribution, Metabolism, Excretion, and Toxicity) profile of the best repurposed FDA-approved NSAID was performed using SwissADME (https://swissadme.ch/) accessed on 20 April 2026. The SMILES notation of the compound was used as input, and key pharmacokinetic properties were analyzed. Lipophilicity was evaluated using multiple models, with a consensus LogP value between 1 and 3 considered optimal for membrane permeability and absorption. Water solubility was assessed, where a LogS value above −4 indicated acceptable solubility for oral drugs. Gastrointestinal (GI) absorption was evaluated, with high absorption considered favorable. Blood–brain barrier (BBB) permeability was predicted, where non-permeability was preferred to minimize central nervous system (CNS) side effects. P-glycoprotein (P-gp) substrate status was also analyzed; non-substrates are favorable to reduce drug efflux and enhance bioavailability. Cytochrome P450 enzyme inhibition was examined for major isoforms (CYP1A2, CYP2C19, CYP2C9, CYP2D6, CYP3A4), where lack of inhibition is preferred to reduce drug–drug interaction risk. Drug-likeness was evaluated based on multiple filters: Lipinski’s Rule of Five (no more than 1 violation), Veber’s rule (≤10 rotatable bonds and polar surface area ≤140 Å^2^), and others such as Ghose, Egan, and Muegge. A bioavailability score of 0.55 or higher was considered indicative of good oral bioavailability.

## 5. Conclusions

This study highlights the potential of an integrated computational drug repurposing approach for identifying new therapeutic applications of existing drugs against antimicrobial resistance targets. Among the evaluated FDA-approved NSAIDs, sulfasalazine emerged as the most promising candidate against OXA-23 β-lactamase based on its favorable binding interactions, molecular stability, and predicted pharmacokinetic characteristics. These findings demonstrate the utility of combining network pharmacology with molecular modeling to prioritize potential inhibitors of carbapenem resistance mechanisms in *Acinetobacter baumannii*. Further experimental investigations, including enzymatic and antibacterial assays, are required to validate the inhibitory activity and therapeutic potential of sulfasalazine.

## Figures and Tables

**Table 1:** Chemical properties of FDA-approved NSAIDs selected for drug repurposing analysis against *blaOXA-23* in *Acinetobacter baumannii*.

Drug Name	Drugbank ID	Pub Chem ID	Molecular Weight	Molecular Formula	Smiles Structure
Aspirin	DB00945	2244	180.16 g/mol	C_9_H_8_O_4_	CC(=O)OC1=CC=CC=C1C(=O)O
Balsalazide	DB01014	54585	357.32 g/mol	C_17_H_15_N_3_O_6_	C1=CC(=CC=C1C(=O)NCCC(=O)O)N=NC2=CC(=C(C=C2)O)C(=O)O
Bromfenac	DB00963	60726	334.16 g/mol	C_15_H_12_BrNO_3_	C1=CC(=C(C(=C1)C(=O)C2=CC=C(C=C2)Br)N)CC(=O)O
Celecoxib	DB00482	2662	381.4 g/mol	C_17_H_14_F_3_N_3_O_2_S	CC1=CC=C(C=C1)C2=CC(=NN2C3=CC=C(C=C3)S(=O)(=O)N)C(F)(F)F
Diclofenac	DB00586	3033	296.1 g/mol	C_14_H_11_Cl_2_NO_2_	C1=CC=C(C(=C1)CC(=O)O)NC2=C(C=CC=C2Cl)Cl
Diflunisal	DB00861	3059	250.2 g/mol	C_13_H_8_F_2_O_3_	C1=CC(=C(C=C1C2=C(C=C(C=C2)F)F)C(=O)O)O
Etodolac	DB00749	3308	287.35 g/mol	C_17_H_21_NO_3_	CCC1=C2C(=CC=C1)C3=C(N2)C(OCC3)(CC)CC(=O)O
Fenoprofen	DB00573	3342	242.27 g/mol	C_15_H_12_O_3_	CC(C1=CC(=CC=C1)OC2=CC=CC=C2)C(=O)O
Flurbiprofen	DB00712	3394	244.26 g/mol	C_15_H_13_FO_2_	CC(C1=CC(=C(C=C1)C2=CC=CC=C2)F)C(=O)O
Ibuprofen	DB01050	3672	206.28 g/mol	C_13_H_18_O_2_	CC(C)CC1=CC=C(C=C1)C(C)C(=O)O
Indomethacin	DB00328	3715	357.8 g/mol	C_19_H_16_ClNO_4_	CC1=C(C2=C(N1C(=O)C3=CC=C(C=C3)Cl)C=CC(=C2)OC)CC(=O)O
Ketoprofen	DB01009	3825	254.28 g/mol	C_16_H_14_O_3_	CC(C1=CC(=CC=C1)C(=O)C2=CC=CC=C2)C(=O)O
Ketorolac	DB00465	3826	255.27 g/mol	C_15_H_13_NO_3_	C1CN2C(=CC=C2C(=O)C3=CC=CC=C3)C1C(=O)O
Meclofenamic acid	DB00939	4037	296.1 g/mol	C_14_H_11_Cl_2_NO_2_	CC1=C(C(=C(C=C1)Cl)NC2=CC=CC=C2C(=O)O)Cl
Mefenamic acid	DB00784	4044	241.28 g/mol	C_15_H_15_NO_2_	CC1=C(C(=CC=C1)NC2=CC=CC=C2C(=O)O)C
Meloxicam	DB00814	54677470	351.4 g/mol	C_14_H_13_N_3_O_4_S_2_	CC1=CN=C(S1)NC(=O)C2=C(C3=CC=CC=C3S(=O)(=O)N2C)O
Nabumetone	DB00461	4409	228.29 g/mol	C_15_H_16_O_2_	CC(=O)CCC1=CC2=C(C=C1)C=C(C=C2)OC
Naproxen	DB00788	156391	230.26 g/mol	C_14_H_14_O_3_	C[C@@H](C1=CC2=C(C=C1)C=C(C=C2)OC)C(=O)O
Nepafenac	DB06802	151075	254.28 g/mol	C_15_H_14_N_2_O_2_	C1=CC=C(C=C1)C(=O)C2=CC=CC(=C2N)CC(=O)N
Oxaprozin	DB00991	4614	293.3 g/mol	C_18_H_15_NO_3_	C1=CC=C(C=C1)C2=C(OC(=N2)CCC(=O)O)C3=CC=CC=C3
Piroxicam	DB00554	54676228	331.3 g/mol	C_15_H_13_N_3_O_4_S	CN1C(=C(C2=CC=CC=C2S1(=O)=O)O)C(=O)NC3=CC=CC=N3
Sulfasalazine	DB00795	5339	398.4 g/mol	C_18_H_14_N_4_O_5_S	C1=CC=NC(=C1)NS(=O)(=O)C2=CC=C(C=C2)N=NC3=CC(=C(C=C3)O)C(=O)O
Sulindac	DB00605	1548887	356.4 g/mol	C_20_H_17_FO_3_S	CC\1=C(C2=C(/C1=C\C3=CC=C(C=C3)S(=O)C)C=CC(=C2)F)CC(=O)O
Suprofen	DB00870	5359	260.31 g/mol	C_14_H_12_O_3_S	CC(C1=CC=C(C=C1)C(=O)C2=CC=CS2)C(=O)O
Tolmetin	DB00500	5509	257.279 g/mol	C_15_H_15_NO_3_	CC1=CC=C(C=C1)C(=O)C2=CC=C(N2C)CC(=O)O
Valdecoxib	DB00580	119607	314.4 g/mol	C_16_H_14_N_2_O_3_S	CC1=C(C(=NO1)C2=CC=CC=C2)C3=CC=C(C=C3)S(=O)(=O)N

## Data Availability

The original contributions presented in this study are included in the article. Further inquiries can be directed to the corresponding author.
